# Interaction and Kinetics Study of the Co-Gasification of High-solid Anaerobic Digestate and Lignite

**DOI:** 10.3390/molecules25030459

**Published:** 2020-01-22

**Authors:** Shengqiang Chang, Zhikai Zhang, Lixia Cao, Liqiang Ma, Fang Wang, Jihui Li, Wangliang Li

**Affiliations:** 1 School of Chemical & Environmental Engineering, China University of Mining & Technology (Beijing), Beijing 100083, China; changshengqiang@yeah.net (S.C.); mlqiang@cumtb.edu.cn (L.M.); 2 The Key Laboratory of Green Process and Engineering, Institute of Process Engineering, Chinese Academy of Sciences, Zhongguancun, Haidian District, Beijing 100190, China; zhang_zk@163.com (Z.Z.); lxcao@ipe.ac.cn (L.C.); wfangwan@163.com (F.W.); 3 Research Institute of Zhejiang University-Taizhou, 618 Shifu Street West, Jiaojiang City, Zhejiang 318000, China

**Keywords:** blending method, co-gasification, digestate, kinetics, interaction

## Abstract

This study aims at investigating the interaction and kinetics behavior of the co-gasification of digestate and lignite. The co-pyrolysis performances of digestate and lignite blended by dry process were better than that blended by wet process, while the wet-blending process could improve the performance in co-gasification stage because of the larger pore diameter and pore volume. When anaerobic digestion (AD) time was 40 days, the synergistic interaction between digestate and lignite were the most remarkable based on the results of thermogravimetric analysis (TG) and the experiments in the lab-scale downdraft fixed bed gasifier. Kinetics study showed that the increase of AD time and the addition of digestate in lignite decreased the activation energy of the co-gasification reaction.

## 1. Introduction

The exhaustion of fossil energy and environmental pollution are becoming the barriers of sustainable social development. Bioenergy with low greenhouse gas emission meets the growing energy demand and plays a critical role in promoting renewable alternatives. Through anaerobic digestion (AD), methane can be generated under ambient conditions from various substrates, such as sewage sludge, food waste, forestry resource, living stock manure, and agricultural waste [1,2]. During the past decades, the Chinese government paid great attention to the development of biogas industry. By the end of 2015, 41.93 million household biogas facilities and 110,975 biogas plants have been built in China, resulting in the significant growth in digestate output, which was mostly used as soil fertilizer [3]. However, the digestate contains high content of harmful substances such as heavy metals, pathogens, trace herbicides and fungicides, which will have potential adverse effects on food safety and ecological environment. With the rapid development of high-solid anaerobic digestion (HSAD), total solid (TS) content higher than 10%, the production rate of digestate has increased dramatically and there is an urgent demand to dispose and reuse the digestate safely.

For the lignocellulosic biomass, only the fractions of cellulose and hemicellulose can be converted in the AD process, and the energy conversion ratio of lignocellulosic biomass is about 33–50% because of its rigid structure and existence of non-biodegradable lignin [4]. Moreover, the lignin content in the digestate is relatively higher than that in the raw biomass feedstock, which is favorable for gasification to realize the complete energy conversion, eliminating the pathogens and immobilization of the heavy metals in the inorganic matrix [5,6,7,8,9,10]. The properties of digestate and its feasibility of gasification were studied by many researchers. Li et al. [11] found that more than 80 wt% of the digestate were volatile matters, which can be used as gasification feedstock to produce syngas. However, digestate has low energy density, which leads to low heating value of produced gas and low-quality products in pyrolysis and gasification. The bio-oil obtained from digestate pyrolysis needs to be further upgraded to improve its quality to overcome high acidity, instability, low heating value, etc. [12]. Wang et al. [13,14] investigated the pyrolysis performances of the corn straw fermentation residues and found that the phenol yield, especially the content of vinyl phenol, increased gradually with the increase of temperature. Although gasification is indisputably considered as a promising and effective technology to dispose digestate, it still encounters many problems, such as low gasification efficiency and low-valued products. 

Co-gasification is widely adopted for the disposal of biomass and clean utilization of coal. It could not only inhibit the generation of SO_x_ and NO_x_, but also reduce the emission of greenhouse gases [7,15,16,17,18]. Coal has the advantages of high energy density and high calorific value, while the combustion of coal may lead to serious environmental pollution. Although the pyrolysis and gasification of coal have been studied for many years, there are still a lot of challenges to overcome. For instance, hydrogen used as gasification agent is needed in the gasification process of coal to produce CH_4_, but the price of hydrogen is expensive [19]. Since the digestate has high H/C ratio, co-gasification of the digestate and coal may enhance the overall energy efficiency if they are blended with appropriate proportion and approach. Yao et al. [20] conducted the co-gasification of digestate and woody chips at different mass ratios and moisture contents. The results showed that when the mass ratio of digestate was 20 wt% and moisture content was 30 wt%, the optimal energy efficiency had reached 70.8%. However, the co-gasification of digestate and coal has been seldom reported. 

To realize the highest reaction activity and the maximum energy recovery, the interaction and the kinetics in co-gasification of digestate and lignite need be investigated to find out a reasonable AD time for co-gasification of digestate and lignite. In addition, the interaction reaction mechanism should be investigated to further optimize the overall energy efficiency of AD and gasification coupling process. Until now, there has been no study that has reported on the interaction and kinetics study of co-gasification of digestate and lignite. 

To explore the interaction between the digestate and lignite in co-gasification process, it is necessary to mix the two feedstocks homogeneously. Many approaches to mix the materials such as using ethanol, incipient wetness impregnation method and physical methods, are used in the field of electrode material preparation, catalytic pyrolysis and raw materials mixing, respectively [21]. Wu et al. [22] investigated the effects of mixing methods on the cellulose-hemicellulose interactions during pyrolysis, blending cellulose and hemicellulose manually with a hydraulic press machine under 20 MPa comparing with native mixtures. Couhert et al. [23] compared the intimate mixing using mortar with simple mix when blending the cellulose, hemicellulose, and lignin during pyrolysis. In addition, the pore structure of digestate biochar is quite different from that of coal char. The blending method may affect the interaction between biochar and catalytic minerals, such as alkali metals and alkaline earth metals, the morphology and covalent linkages between digestate and coal. Thus, the blending method plays a significant role in affecting the performances of co-pyrolysis and co-gasification. The effect of different blending methods on the properties of co-pyrolysis and co-gasification was investigated in detail. The effects of different digestion treatment time on the interaction and kinetics of co-pyrolysis and co-gasification were also explored by TG. The co-gasification experiments of digestate and lignite were conducted in a lab-scale downdraft fixed gasifier. Moreover, the kinetic models such as three-dimensional diffusion, nucleation and growth models were employed by using Coats–Redfern method in order to observe the optimum mechanisms for the thermal conversion process to describe the reactive behavior and to determine the kinetic parameters. 

## 2. Results and Discussion

### 2.1. Effect of Blending Methods on Co-pyrolysis and Co-gasification

**Figure 1:**
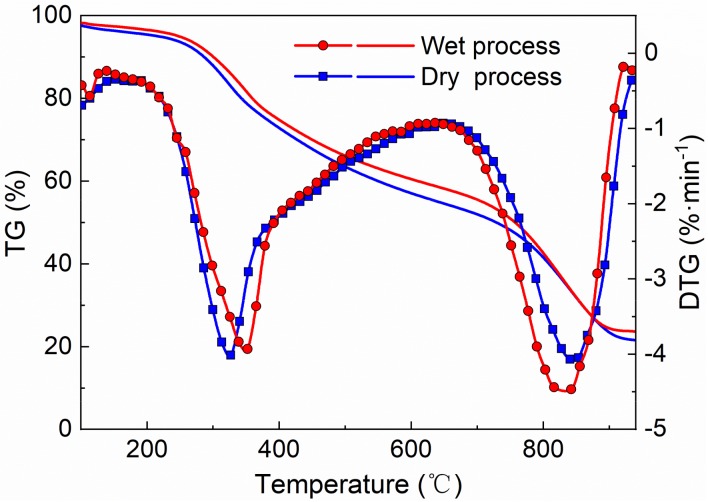
TG and DTG curves of the lignite and digestate mixtures blended by wet and dry process.

Digestate and lignite were blended with the ratio of 50:50% (*wt*/*wt*). Then, the effects of blending methods on thermal conversion were investigated using TG. As shown in Figure 1, the reaction process can be subdivided into two stages: pyrolysis (stage 1, S1) and gasification (stage 2, S2). As the temperature increasing from 200 °C to 650 °C, the decomposition and emission of volatiles happened in the first stage. In the second stage, gasification of biochar took place with the temperature ranging from 700 °C to 950 °C. For the sample blended by dry process, the values of the derivative curve (DTG) of TG curve in stage 1 were slightly higher than that blended by wet process and the T_max_ was lower than that of the sample blended by wet process, which indicated that the dry-blending process can enhance the co-pyrolysis greater than wet-blending process. On the other hand, the values of DTG curve of dry process in stage 2 were lower than that blended by wet process, which indicated that the wet process will promote co-gasification greater than dry-blending process.

**Figure 2:**
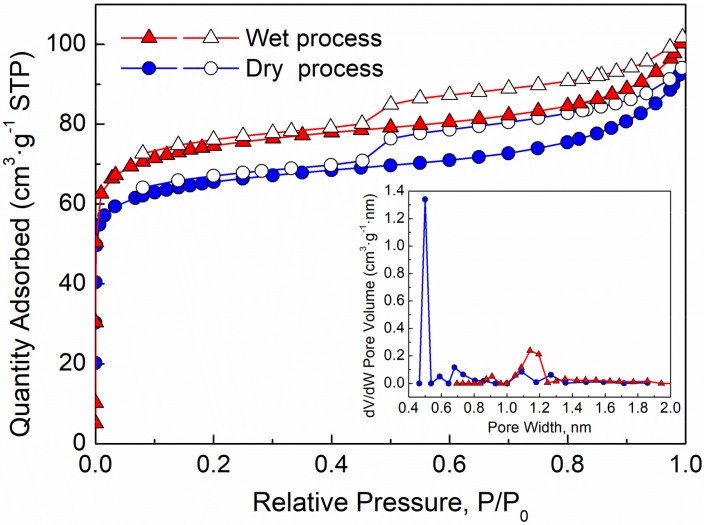
N_2_ adsorption-desorption isotherms and pore size distribution of the biochar.

The adsorption-desorption isotherms and pore size distribution of biochar samples are presented in Figure 2. According to IUPAC, the adsorption/desorption isotherms of biochar samples presented the resemble features between the type I and II. The hysteresis loops of the biochar belong to the type H4, indicating the rich microporous structures. The adsorption-desorption isotherms of the both biochar samples showed a sharp knee at P/P_0_ around 0.01, indicating the narrow pore diameter distribution [24]. The adsorption capacity of biochar samples was quantified by the amount of adsorbed nitrogen [25]. When the P/P_0_ is less than 0.01, the micropores started to be filled quickly and the adsorption capacity of sample blended by wet process was higher than that of biochar sample blended by dry process, indicating the content of micropores and pore volume in biochar sample blended by wet process were more than that of biochar samples blended by dry process. The pore sizes of the biochar samples were smaller than 2 nm, indicating that the pores in the biochar were mainly micropores. Moreover, compared with blending by dry process, blending by wet process increased the number and pore diameter of micropores.

The surface area, pore volume and average pore diameter parameters of biochar samples under different blending methods are shown in Table 1. Compared to dry process, the specific surface area, pore volume and average pore diameter of samples by wet process increased by 14.69%, 19.23%, and 32.00%, respectively. Ping et al. [24,26] reported that micropore was the main contribution to the surface area, and the results confirmed that the amount of micropores in the biochar sample blended by wet process was larger than that blended by dry process. It was found that blending lignite and digestate by wet process can promote the formation of pores in the biochar dramatically, accelerating the co-gasification of the mixture. Digestate and lignite were rich in organic volatile matters. Ethanol, used as organic solvents, can break the bridging of organic matters and dissolve the samples partially [27]. Therefore, when blending the samples by wet process, part of the organic matters was extracted from the inside of the biomass and enriched outside of the sample particles. It would leave spaces among the various components in the material and the links of interaction among the various components disappeared. As a result, the catalytic ingredients in the materials blended by wet process were not able to play a catalytic role, which lead to the inhibition of the pyrolysis reaction in stage 1 comparing with dry process, while blending by dry process made the components more intimate and strengthened the interactions among the different components in stage 1. Although blending by wet process would promote the performance of co-gasification, the improvement is not obvious comparing with blending by dry process. Therefore, the following experimental samples were mixed by dry process.

### 2.2. TG Analysis of Digestate and Lignite

**Figure 3:**
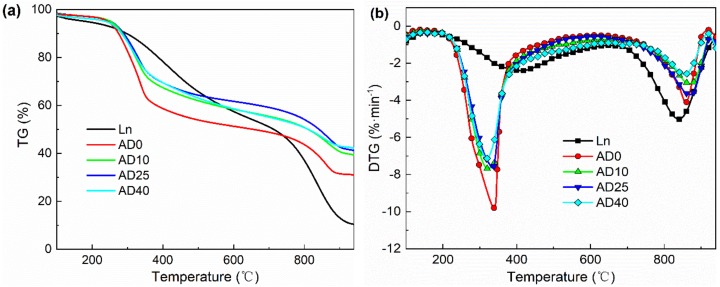
(**a**) TG and (**b**) DTG curves of lignite and AD digestate samples.

The experimental TG/DTG curves of lignite and digestate are shown in Figure 3a,b, respectively. It can be seen that the ash content of digestate was obviously higher than that of lignite, and the ash content of digestate increased with the increase of AD time, which was in accordance with the results of the proximate analysis shown in Table 2. From the DTG curves, it can be observed that there were two reaction stages occurring in sequence. The decomposition of volatiles and emission of gaseous species took place in stage 1 with the temperature ranging from 200 °C to 400 °C. The pyrolysis temperature of digestate was much lower than that of the lignite, which ranged from 210 °C to 650 °C, meaning that digestate could be pyrolyzed more easily than the lignite at lower temperature. In the next stage, biochar gasification reactions took place with the temperature ranging from 650 °C to 950 °C. The gasification temperature of digestate was higher than that of lignite, which ranged from 700 °C to 935 °C, indicating that the lignite could be gasified earlier than digestate. Li et al. [11] investigated the effect of mass ratio of grass and chicken manure on the digestate TG. The results showed that the volatile matters in digestate increased and the contents of ashes and fixed carbons decreased with the increase of grass contents. Because the anaerobic sludge brought inorganic non-flammable salts and sands into the mixture, the ash content of digestate was higher than the grass.

The pyrolysis and gasification performances (the maximum rates of the weight loss (DTG_max_), and corresponding maximum temperature (T_max_)) of lignite, digestate and mixtures were calculated from the TG results, as shown in Table 3. The highest and the lowest DTG_max_ for single samples among digestate in stage 1 were 9.87%·min^−1^ for AD0 and 7.22%·min^−1^ for AD40. The highest and the lowest DTG_max_ for single samples among digestate in stage 2 were 4.19%·min^−1^ for AD0 and 2.62%·min^−1^ for AD40, respectively. Obviously, the DTG_max_ of digestate decreased with the increase of AD time in the pyrolysis and gasification stages in spite that the DTG_max_ of AD25 was slightly higher than that of AD10 in stage 2, indicating that the reactivity of digestate decreased as AD time continued, and the DTG_max_ in the pyrolysis stage was significantly higher than that in the gasification stage. On the other hand, for lignite, the trend was opposite to that of digestate, and its DTG_max_ in gasification stage was higher than that in pyrolysis stage, which was similar to the Xu’s study in that the DTG_max_ of lignite was higher than that in pyrolysis stage and the trend of biomass was opposite to the lignite [28].

For the mixture with the mass ratio of lignite to digestate 50:50% (*wt*/*wt*), Ln-AD0 and Ln-AD40 showed the highest DTG_max_ value of 5.22%·min^−1^ and 4.26%·min^−1^ in stage 1 and 2. Whereas, Ln-AD40 had the lowest DTG_max_ value of 3.75%·min^−1^ in stage 1 and Ln-AD25 had the lowest DTG_max_ value of 4.11%·min^−1^ in stage 2, respectively.

The digestate reactivity (*R_m_*) of AD0, AD10, AD25, and AD40, computed by Equation (1), was 3.41%·(min·°C)^−1^, 2.79%·(min·°C)^−1^, 2.80%·(min·°C)^−1^, and 2.54%·(min·°C)^−1^, respectively. The values were higher than that of lignite, 1.17%·(min·°C)^−1^. AD0 has the highest reactivity among all the digestates. The reactivity of Ln-AD0, Ln-AD10, Ln-AD25, and Ln-AD40 was 2.07%·(min·°C)^−1^, 1.69%·(min·°C)^−1^, 1.74%·(min·°C)^−1^, and 1.63%·(min·°C)^−1^, respectively. Because of the different AD times, the contents of volatiles in the digestates varied. Therefore, the AD0, which underwent AD for the shortest time, exhibited the highest reactivity.

### 2.3. Analysis of Interaction between Digestate and Lignite

**Figure 4:**
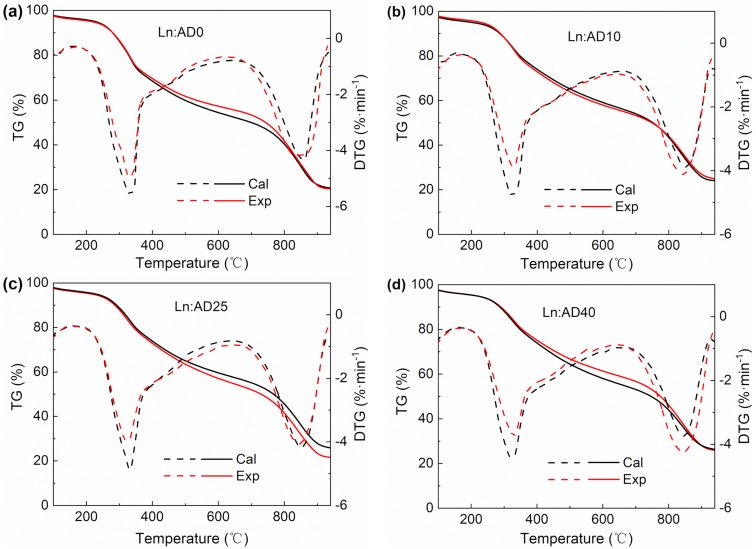
Experimental and the calculated TG, DTG curves of lignite and digestate with (**a**) AD 0 day, (**b**) AD 10 days, (**c**) AD 25 days and (**d**) AD 40 days at the ratio of 50:50% (*wt*/*wt*).

Equations (2) and (3) were used to calculate the theoretical DTG curve and to identify whether there were interactions in stage 1 and stage 2. Figure 4 illustrated the comparison between the experimental and the calculated TG and DTG curves of lignite and digestate with the mass ratio 50:50% (*wt*/*wt*). It can be seen that the AD time had great influences on the performances of the co-pyrolysis and co-gasification. For stage 1, the DTG values obtained from experiments were lower than that from theoretical calculation for all the mixtures, indicating that the addition of the digestate inhibited the co-pyrolysis reaction. For stage 2, the values obtained from DTG were higher than from theoretical calculation for AD10 and AD40, which indicated that the addition of the digestate promoted the co-gasification reaction. For AD0 and AD25, the DTG experiment results were close to the theoretical calculation. It was hardly to judge whether the addition of AD0 and AD25 in lignite can promote the reaction in stage 2.

**Figure 5:**
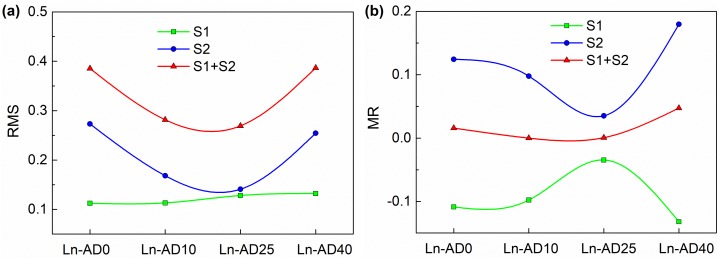
(**a**) RMS and (**b**) MR of co-pyrolysis and gasification of lignite and digestate with different AD times.

To quantify the interaction between digestate and lignite in stage 1 and stage 2, two parameters, Root Mean Square (RMS) and MR, were calculated according to the Equations (4) and (5). The RMS of co-pyrolysis and co-gasification of lignite and digestate with different AD times is shown in Figure 5a. In stage 1, with the increase of AD time, the RMS increased slightly and then, remained constant gradually. In stage 2, the RMS firstly decreased and then, increased with the increase of AD time. The RMS of Ln-AD0 and Ln-AD40 were higher than that of other samples, indicating that the synergistic interaction of the digestate and lignite were the most remarkable. The MR of co-pyrolysis and co-gasification of lignite and digestate with different AD times is shown in Figure 5b. It can be seen that all the MR values for stage 1 were less than zero, indicating the interactions between lignite and digestate were negative. On the other hand, the MR values for stage 2 were higher than zero indicating the interactions among the all mixtures during the co-gasification were positive. This agreed well with the trend of experimental and calculated values of DTG curves. The interaction between lignite and digestate of 40 d were the most remarkable because it has the most prominent RMS and MR values. Digestate mainly consisted of the degraded corn straw, cow dung, and sludge. Corn straw and cow dung contained high content of cellulose, hemicellulose, and lignin. The cellulose and lignin content had significant impact on the co-pyrolysis. The lignin can inhibit the pyrolysis of cellulose [29]. Therefore, the existence of cellulose and lignin may have a negative impact on the co-pyrolysis of lignite and digestate. Besides cellulose hemicellulose and lignin, the substrate also contained some protein and lipid, which had the characteristics of rich fat structure, long fat chain and low bond energy. During the gasification process, the protein and lipid were easy to break and form abundant free radicals and volatiles. Free radicals not only reacted with organic matters, but also participated in the reaction of lignite, thus, promoting the gasification reaction [30]. For the AD time of 0 d, the content of organic matters was the highest. Therefore, the synergistic interaction of gasification was remarkable. With the increase of AD time, more organic matters could be hydrolyzed and consumed in AD. After being digested for 10 d, the synergistic interaction between lignite and digestate weakened relatively. Moreover, as the AD reaction going on, more hydrolyzed biomass participated in AD and the structure was broken, resulting in more porous surface structure of pyrolysis biochar, which was favorable to the gasification reaction and the diffusion of gasification products. Therefore, the synergistic interaction in co-gasification were obviously enhanced when the mixture of AD40 and lignite was used as feedstock. As the AD time continued to increase, the synergistic interaction in co-gasification would continue to be enhanced.

In addition, digestate and lignite were influenced by the catalytic effects of alkali metals and alkaline earth metals in the whole reaction process, which enhanced the thermal conversion performances. Alkali metals and alkaline earth metals in ash, such as Ca, K, can significantly promote the thermal conversion reaction in the pyrolysis and gasification process. Edreis [31,32] reported that the mixture of the petroleum coke and biomass wastes had high reactivity because of the catalytic effects of alkali metals in the mixture. Fe_2_O_3_ also played a catalytic role during the pyrolysis of sewage sludge because Fe_2_O_3_ enhanced the evaporation of the volatile and promoted the crack of biochar [33].

### 2.4. Co-Gasification of Digestate and Lignite in a Lab-Scale Downdraft Fixed Bed Gasifier

**Figure 6:**
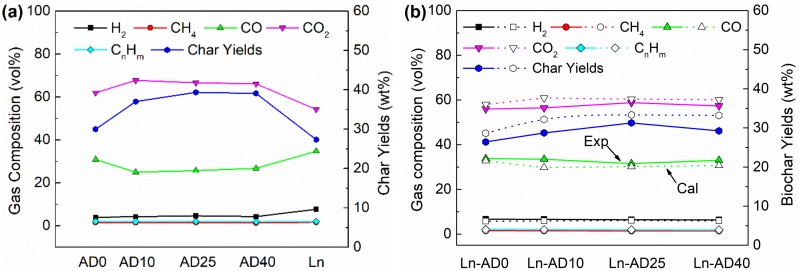
The gas compositions and biochar yields of (**a**) gasification of single samples and (**b**) co-gasification of lignite and digestate.

The gasification experiments were conducted in a lab-scale downdraft fixed bed gasifier at 950 °C. Figure 6a showed the gas compositions and biochar yields of single samples. The char yield of lignite was lower than the all digestate indicating that the ash content of lignite was lower than that of digestate, which was consistent with TG experiments. The main gas products of digestate and lignite were CO, CO_2_, CH_4_, H_2_, and C_n_H_m_. The CO contents of AD10, AD25, and AD40 were lower than AD0, indicating part of organic matter was consumed during the anaerobic digestion. However, the CO contents increased lightly with the increasing of anaerobic digestion time from 10 days to 40 days. The reason may be that the surface of biomass was hydrolyzed and the structure was broken, resulting porous surface, which was in favor for the gasification. 

Figure 6b presented the gasification experimental results of mixture that the digestate and lignite was blended as mass ratio 50:50% (*wt*/*wt*). The calculated values of gas compositions and char yields were based on the single samples according to the Equation (2). The CO contents of experiments were higher than the calculated values. Moreover, the experimental values of CO_2_ content and char yields were lower than the calculated values, increasing the yield of CO and improving the CO_2_ consumption. It indicated that the synergistic interaction of digestate and lignite occurred in the co-gasification process. The experimental value of Ln-AD40 biochar yield, which compared with calculation values, reduced the greatest, 11.89%, among the four mixtures from Figure 6b. This means that the synergistic interaction of Ln-AD40 in the gasification process was the most remarkable, which was consistent with the interaction analysis results of TG in Section 2.3. According to Hu’s study, a part of alkali metal K migrated from biomass to coal char surface, while a part of alkali-earth metal Ca was transferred from coal to the biomass char surface in the co-gasification, leading to the synergy interaction of biomass and coal [34]. The metal migration between lignite and digestate in their co-gasification may be the reason that resulted in the synergy interaction in co-gasification of digestate and lignite. 

### 2.5. Kinetic Analysis

The kinetic parameters including activation energy *E*, pre-exponential factor *A*, and correlation coefficients *R^2^* for different samples are shown in Table 4 and Table 5, respectively. It can be seen that all correlation coefficients of each experimental sample in the two reaction stages were approximately 1, which showed that the corresponding reaction models fitted the experimental results well.

In stage 1, the activation energy of lignite and digestate with the mass ratio of 50:50% (*wt*/*wt*) was lower than that of digestate, but higher than that of lignite. According to the calculation by model *A_0.5_*, the activation energy of lignite in stage 2 was similar to the Xu’s study that the activation energy of lignite gasification was 183.90 kJ·mol^−1^ [28]. According to the results of model *D_3_*, *D_4_,* and *A_0.5_*, the activation energy of digestate in stage 1 decreased gradually from 96.95 kJ·mol^−1^, 93.55 kJ·mol^−1^, and 102.19 kJ·mol^-1^ at an AD time of 0 d to 81.46 kJ·mol^−1^, 78.70 kJ·mol^−1^, and 85.69 kJ·mol^−1^ at an AD time of 40 d with the increase of AD time. For the samples with the same AD time, the activation energy of digestate in stage 2 decreased gradually from 120.01 kJ·mol^−1^ and 74.82 kJ·mol^−1^ at an AD time of 0 d to 81.14 kJ·mol^−1^ and 47.41 kJ·mol^−1^ at an AD time of 40 d, calculated by model *D_3_* and *D_4_*, respectively. However, when calculated by model *A_0.5_*, the activation energy increased slightly for the sample with an AD time of 40 d compared to the sample with an AD time of 25 d in stage 2. The activation energy of lignite and digestate blended with the blending ratio of 50:50% (*wt*/*wt*) in stage 1 decreased gradually from 65.93 kJ·mol^−1^, 64.18 kJ·mol^−1^, and 68.59 kJ·mol^−1^ at an AD time of 0 d to 55.07 kJ·mol^−1^, 53.73 kJ·mol^−1^, and 57.12 kJ·mol^−1^ at an AD time of 40 d, obtained by model *D_3_*, *D_4_*, and *A_0.5_*, respectively. The activation energy of the mixtures with ratio of 50:50% (*wt*/*wt*) in stage 2 also decreased gradually with the increase of AD time from 119.82, 82.33, and 192.37 kJ·mol^−1^ at an AD time of 0 d to 99.15 kJ·mol^−1^, 73.18 kJ·mol^−1^, and 145.73 kJ·mol^−1^ at an AD time of 40 d, obtained by model *D_3_*, *D_4_,* and *A_0.5_*, respectively. The activation energy of lignite and digestate of 40 d was the lowest, which was consistent with the results of TG and co-gasification in the lab-scale gasifier.

High activation energy means that the reactions needs higher temperature or longer reaction time [35]. The activation energy decreased with the increase of AD time, and the addition of digestate in lignite can significantly reduce the activation energy. As shown in Table 2, the ash content of digestate increased with the increase of AD time. The alkali metals and alkaline earth metals cannot be consumed in AD process, leading to the more alkali metals and alkaline earth metals in the digestate with the increase of AD time, which played a catalytic role in the pyrolysis and gasification [31]. Hence, the catalytic effect was becoming more and more obvious with the increase of AD time, resulting in the reduction of activation energy gradually.

## 3. Materials and Methods

### 3.1. Feedstock Materials

The selected coal samples were collected from Xiaolongtan lignite (Ln). The digestate was produced from HSAD of corn straw, cattle manure and sludge in lab-scale AD reactors. The corn straw was crushed less than 3 cm after air dried. The corn straw, sludge, cattle manure, and water were blended as mass ratio 1.13:3.65:6.39:1 and the total weight of mixture was 7.00 kg. The AD conditions were as follows: total solid 30% and ambient temperature 35 ± 1 °C. The mixture samples were digestated for 0 day, 10 days, 25 days, and 40 days, and denoted as AD0, AD10, AD25, and AD40, respectively. The digestate samples were dried at 105 °C for 24 h, ground and screened below 200 mesh. The ultimate and proximate analysis of digestate and lignite are shown in Table 2. The ash compositions of digestate and lignite are presented in Table 6.

### 3.2. Experimental Set-Up

The effects of two different blending methods (by wet process and by dry process) on the TG experiments of mixtures were investigated with mass ratio of digestate to lignite 50:50% (*wt*/*wt*). That the ethanol was used as dispersing medium to mix the digestate and lignite was defined as wet process. The dry process used mortar to mix the different samples.

Secondly, four kinds of digestate were blended with lignite with the ratio of 50:50% (*wt*/*wt*). TG experiments were carried out to investigate the interaction of lignite and digestate under different digestion times blended by optimal method. 

Afterwards, the co-gasification experiments were carried out in the downdraft fixed bed gasifier to investigate whether the co-gasification can improve the performance. 

Finally, the reaction kinetics of co-pyrolysis and co-gasification were explored under different conditions. The experiments contained three repetitions.

### 3.3. Blending Methods

#### 3.3.1. The Wet Process and Dry Process

For wet process, 1.00 g digestate and 1.00 g lignite were blended at the ratio of digestate to lignite 50:50% (*wt*/*wt*) in 50 mL ethanol (≥ 99.7%, Beijing Chemical Works) in a 150 mL beaker. The mixture was stirred for 30 min at 350 r·min^−1^, and then, placed for 24 h. After the ethanol was volatilized, the beaker was placed in the oven at 105 °C for 24 h. The samples were ground into powder. For dry process, 1.00 g of digestate and 1.00 g of lignite were poured into the mortar for complete blending. 

#### 3.3.2. Preparation and Pore Structure Analysis of Pyrolysis Biochar

To investigate the influence of blending methods on the co-pyrolysis and co-gasification performances, the mixtures of the digestate and lignite were prepared according to the Section 3.3.1. Then, 2.00 g samples were pyrolyzed to produce biochar in a tubular furnace. Pure N_2_ was used as carrier gas and preloaded for 2 min. The tubular furnace was vacuumed and purged with N_2_ for three times. Finally, the flow rate was set at 100 mL·min^−1^. The temperature of the tube furnace rose from room temperature to 950 °C at 15 °C·min^−1^ and kept for 1 h. The pore structures of pyrolysis biochar prepared by two blending methods were characterized. 

Nitrogen adsorption experiments (temperature, 77 K) were conducted using physical adsorption analyzer (Micromeritics ASAP 2020HD88). The surface area and average pore diameter of biochar samples are measured using Brunauer–Emmett–Teller (BET). The pore volume is calculated from the t-plot method.

### 3.4. TG Analysis of Digestate and Lignite

#### 3.4.1. TG Experiments

Non-isothermal co-gasification experiments of digestate and lignite were carried out by TG analysis (Setaram Labsys Evo, Lyon, Rhône Province, France). Pure CO_2_ was introduced in the reactor as gasifying agent. Temperature was risen from room temperature to 950 °C at 15 °C·min^−1^.

#### 3.4.2. Reactivity Measurements

The reactivity of pyrolysis and gasification reactivity was calculated with the following [36,37]:

(1) $$R_{m}=100\sum \left({\mathrm{DTG}}_{max}/T_{max}\right)$$

where *R_m_* is the reactivity (%·(min·°C)^−1^), DTG_max_ is the maximum mass loss rate (%·min^−1^), and T_max_ is the maximum temperature, correspondingly (°C).

### 3.5. Analysis of Interaction Between Digestate and Lignite

The TG/DTG theory values of the co-gasification of lignite and digestate with different AD times are calculated according to the Equations (2) and (3) [35,38]. By comparing the theoretical and experimental TG/DTG results, it could be concluded whether there is synergistic interaction during the co-pyrolysis and co-gasification of lignite and digestate:

(2) $$w=x_{D}{\left(w\right)}_{D}+x_{L}{\left(w\right)}_{L}$$

(3) $$dw/dt=x_{D}{\left(dw/dt\right)}_{D}+x_{L}{\left(dw/dt\right)}_{L}$$

where w is the weight loss (%), gas composition (vol%), and char yields (wt%), dw/dt is the weight loss rate (%·min^−1^), and $x_{D}$ and $x_{L}$ correspond to the mass ratio of digestate to lignite, respectively.

In order to quantitatively evaluate the interaction of co-pyrolysis and co-gasification, two parameters were used to characterize the reaction. One is the RMS to judge whether there is interaction between digestate and lignite. However, it cannot analyze whether the interaction is positive or negative. Another parameter, MR, is defined as the ratio of average absolute error to average calculated value. Positive MR indicates that fractions of the mixture promotes each other in the reaction. On the contrary, if MR is negative, the interaction is inhibited [30,39].

(4) $$\mathrm{RMS}={\left(\overset{n}{\underset{i=1}{\sum }}{\left(\left(x_{exp}^{i}-x_{cal}^{i}\right)/x_{cal}^{i}\right)}^{2}/n\right)}^{1/2}$$

(5) $$\mathrm{MR}=(\overset{n}{\underset{i=1}{\sum }}\left(x_{exp}^{i}-x_{cal}^{i}\right))/n/x_{cal}^{mean}$$

### 3.6. Co-gasification of Digestate and Lignite in a Lab-scale Gasifier

**Figure 7:**
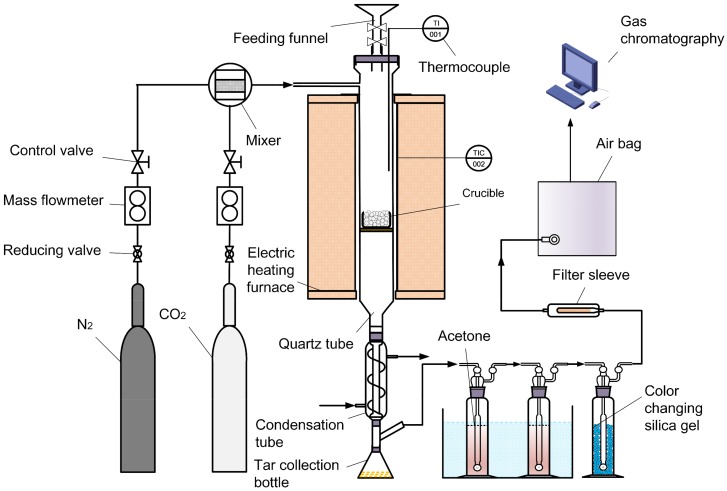
Flow chart of gasification system.

The gasification experiments of digestate and lignite were conducted in a lab-scale downdraft fixed bed gasifier as shown in Figure 7. The internal diameter of quartz tube is 35 mm and the distributor plate is located in the middle of quartz tube. A crucible is placed on distributor plate to store the digestate and lignite.

For each experiment, the crucible was stored with 2.0 g feedstock in the downdraft fixed bed gasifier. The CO_2_ gas (99.99%) was selected as the gasification agent and the gas flow rate was 60 mL·min^−1^ in the gasification process. The gasifier was heated from room temperature to 950 °C at the rate of 50 °C·min^−1^ and stayed the same temperature for a certain time. The gas bag was used to collect the product gas, the composition of which was analyzed by gas chromatography.

### 3.7. Kinetics Study

The kinetics analysis of co-pyrolysis and co-gasification was carried out. The conversion rate is expressed with the following [41]:

(6) $$d\alpha /dT=A\left(-E/RT\right){\left(1-\alpha \right)}^{n}/\beta$$

(7) $$\alpha =\left(m_{0}-m_{t}\right)/\left(m_{0}-m_{\infty }\right)$$

where *α* is the conversion ratio (%), *T* is the absolute temperature (*K*), *A* is the pre-exponential factor (min^−1^), *β* is the constant heating rate (*K*·min^−1^), with *R* = 8.314 J·mol^−1^·*K*^−1^, and *m* is the mass of sample (g). Using Coats-Redfern integral method, the weight loss Equation (6) are fitted and calculated as follows [42,43]:

(8) $$\ln\left[-\ln\left(1-\alpha \right)/T^{2}\right]=\ln\left[AR\left(1-2RT/E\right)/\left(\beta E\right)\right]-E/\left(RT\right),\;\;n=1$$

(9) $$\ln\left[-\ln\left(1-\alpha \right)/T^{2}/\left(1-n\right)\right]=\ln\left[AR\left(1-2RT/E\right)/\left(\beta E\right)\right]-E/\left(RT\right),\;\;n\neq 1$$

Because 1−2RT/E≈1, $\ln\left[AR\left(1-2RT/E\right)/\left(\beta E\right)\right]$ is close to a constant [44]. Suppose Y is $\ln\left[-\ln\left(1-\alpha \right)/T^{2}/\left(1-n\right)\right]$ or $\ln\left[-\ln\left(1-\alpha \right)/T^{2}\right]$, and Y = ax + b. The activation energy *E* and pre-exponential factor *A* of the reaction can be obtained through the values of slope and intercept. In this study, the reaction mechanism function (g(x)) used for the calculation are shown in Table 7 [40]. The mechanism functions *D_3_* and *D_4_* are attribute to a three-dimensional diffusion model. The *D_3_* and *D_4_* belong to the Jander equation and Ginstling-Brounshtein equation, respectively. The mechanism function *A_0.5_* is belong to Avrami-Erofeev equation and the power exponent n is 0.5, which is attribute to randomly nucleating and nucleus growth model. 

## 4. Conclusions

The dry-blending process can improve the reactivity during co-pyrolysis, while the wet-blending process could promote the co-gasification because of the improvement of the pore diameter and pore volume. The thermal conversion of the digestate, lignite and their mixtures occurred in two reaction stages, pyrolysis and gasification. The synergistic interaction occurred in the co-gasification, and not in the co-pyrolysis. Based on the TG results and the co-gasification experiments in the downdraft fixed bed, the synergistic interaction was the most remarkable when the sample of AD40 and lignite was mixed as mass ratio 50:50% (*wt*/*wt*). Three repeated experiments showed consistent results. From the results of kinetic study, the Avrami-Erofeev equation *A*_0.5_, belonging to the randomly nucleating and nucleus growth model, was found to be the most suitable for the whole co-gasification process. The activation energy of the mixture decreased sharply from 192.37 kJ·mol^−1^ to 145.73 kJ·mol^−1^ with an increase of AD time. The co-gasification was found to be a promising way for energy recovery from digestate waste and lignite.

## Figures and Tables

**Table 1:** Textural structure of the char samples blended by dry and wet process.

	Dry Process	Wet Process
Surface area (m^2^·g^−1^)	249.90	286.60
Pore volume (cm^3^·g^−1^)	0.0723	0.0862
Average pore diameter (nm)	0.50	0.66

**Table 2:** Ultimate and proximate analysis of the digestate and lignite.

	Ultimate Analysis (wt%, ad)					Proximate Analysis (wt%, d)		
	C	H	N	S	O^a^	Volatile matter	Ash	Fixed carbon
Lignite	52.04	4.66	1.48	1.31	25.92	54.01	12.09	33.89
AD0	32.33	4.52	1.63	0.35	28.17	56.12	31.88	12.00
AD10	31.09	4.09	2.24	0.43	24.03	50.65	37.42	11.94
AD25	29.73	3.72	2.41	0.51	21.53	47.09	41.41	11.50
AD40	29.64	3.61	2.38	0.54	20.31	47.75	41.48	10.77

^a^ Calculated by difference.

**Table 3:** Gasification performances of the lignite, digestate, and their mixtures.

Characteristics		Ln	AD0	AD10	AD25	AD40	Ln-AD0	Ln-AD10	Ln-AD25	Ln-AD40
S1	T_max_, °C	417	337	331	332	323	334	328	325	334
	DTG_max_, %·min^−1^	2.41	9.87	8.05	7.83	7.22	5.22	3.92	4.06	3.75
S2	T_max_, °C	842	864	871	871	857	831	845	845	842
	DTG_max_, %·min^−1^	5.02	4.19	3.15	3.79	2.62	4.21	4.15	4.11	4.26

T_max_: The temperature (°C) when weight loss rate attained the maximum; DTG_max_: The maximum rate of weight loss. Ln-AD0, Ln-AD10, Ln-AD25, Ln-AD40: The blending ratio of lignite and digestate was 50:50% (*wt*/*wt*).

**Table 4:** The kinetic parameters of single samples.

		S1			S2		
		*E* (kJ·mol^−1^)	*A* (min^−1^)	*R^2^*	*E* (kJ·mol^−1^)	*A* (min^−1^)	*R^2^*
Ln	*D_3_*	40.29	1.62 × 10^0^	0.9985	118.36	1.63 × 10^4^	0.9821
	*D_4_*	38.89	1.15 × 10^0^	0.9980	86.16	1.35 × 10^2^	0.9906
	*A_0.5_*	42.42	2.48 × 10^1^	0.9990	177.68	1.15 × 10^9^	0.9569
AD0	*D_3_*	96.95	2.71 × 10^6^	0.9906	120.01	2.12 × 10^4^	0.9614
	*D_4_*	93.55	1.18 × 10^6^	0.9888	74.82	6.34 × 10^1^	0.9750
	*A_0.5_*	102.19	8.73 × 10^7^	0.9926	208.65	1.02 × 10^10^	0.9385
AD10	*D_3_*	90.31	4.85 × 10^5^	0.9861	102.91	7.27 × 10^2^	0.9833
	*D_4_*	87.17	2.27 × 10^5^	0.9841	61.87	5.37 × 10^0^	0.9883
	*A_0.5_*	95.12	1.40 × 10^7^	0.9886	184.20	7.09 × 10^7^	0.9671
AD25	*D_3_*	87.17	2.26 × 10^5^	0.9911	83.95	4.01 × 10^3^	0.9744
	*D_4_*	84.29	1.12 × 10^5^	0.9899	59.58	2.59 × 10^1^	0.9842
	*A_0.5_*	91.58	5.92 × 10^6^	0.9924	127.05	4.36 × 10^8^	0.9531
AD40	*D_3_*	81.46	8.14 × 10^4^	0.9939	81.14	2.28 × 10^2^	0.9844
	*D_4_*	78.70	4.11 × 10^4^	0.9930	47.41	2.19 × 10^0^	0.9870
	*A_0.5_*	85.69	2.07 × 10^6^	0.9948	147.28	1.02 × 10^7^	0.9736

**Table 5:** The kinetic parameters of lignite and digestate with different AD times.

		S1			S2		
		*E* (kJ·mol^−1^)	*A* (min^−1^)	*R^2^*	*E* (kJ·mol^−1^)	*A* (min^−1^)	*R^2^*
Ln-AD0	*D_3_*	65.93	1.32 × 10^3^	0.9939	119.82	1.65 × 10^4^	0.9739
	*D_4_*	64.18	8.50 × 10^2^	0.9935	82.33	1.29 × 10^2^	0.9913
	*A_0.5_*	68.59	2.32 × 10^4^	0.9942	192.37	1.24 × 10^9^	0.9330
Ln-AD10	*D_3_*	56.78	1.43 × 10^2^	0.9931	112.26	7.03 × 10^3^	0.9799
	*D_4_*	55.26	9.67 × 10^1^	0.9926	76.76	6.77 × 10^1^	0.9925
	*A_0.5_*	59.10	2.31 × 10^3^	0.9938	180.13	3.18 × 10^8^	0.9475
Ln-AD25	*D_3_*	55.55	5.11 × 10^2^	0.9886	110.18	5.52 × 10^3^	0.9751
	*D_4_*	54.20	3.47 × 10^2^	0.9883	75.30	5.68 × 10^1^	0.9905
	*A_0.5_*	57.61	8.25 × 10^3^	0.9890	176.82	2.18 × 10^8^	0.9402
Ln-AD40	*D_3_*	55.07	9.34 × 10^1^	0.9948	99.15	1.42 × 10^3^	0.9586
	*D_4_*	53.73	6.59 × 10^1^	0.9947	73.18	4.22 × 10^1^	0.9766
	*A_0.5_*	57.12	1.42 × 10^3^	0.9950	145.73	5.65 × 10^6^	0.9284

**Table 6:** X-ray fluorescence (XRF) analysis of the ash components of lignite and digestate (wt%).

	CaO	SiO_2_	SO_3_	Al_2_O_3_	Fe_2_O_3_	MgO	P_2_O_5_	K_2_O	Total
Lignite	32.10	21.43	20.14	12.00	7.40	4.64	—	0.74	98.45
Digestate	23.72	23.99	3.51	4.54	2.23	9.95	21.24	5.51	94.69

**Table 7:** Typical kinetic model functions expressions of g(x) and f(x) for solid-state reactions [40].

Model.	Symbol	$g\left(x\right)$	$f\left(x\right)$
Three-dimensional diffusion (Jander)	*D_3_*	${\left[1-{\left(1-x\right)}^{1/3}\right]}^{2}$	$3{\left(1-x\right)}^{2/3}/\left[2-2{\left(1-x\right)}^{1/3}\right]$
Three-dimensional diffusion (Ginstling-Brounshtein)	*D_4_*	$1-2x/3-{\left(1-x\right)}^{2/3}$	$3/\left[2{\left(1-x\right)}^{-1/3}-2\right]$
Nucleation and growth (Avrami-Erofeev)	*A_0.5_*	${\left[-\ln\left(1-x\right)\right]}^{2}$	$\left(1-x\right)/\left[-2\ln\left(1-x\right)\right]$
